# An evaluation of autologous tumor-reactive TIL generation from head and neck squamous cell cancers

**DOI:** 10.1186/2051-1426-3-S2-P37

**Published:** 2015-11-04

**Authors:** Tarsem Mougdil, Christopher Paustian, Zipei Feng, Rom Leidner, Christopher Dubay, Brendan D Curti, Hong-Ming Hu, Walter Urba, Carlo Bifulco, Bernard Fox, Brian Bell

**Affiliations:** 1 Robert W Franz Cancer Research Center, Portland, OR, USA; 2 Earle A. Chiles Research Institute, Portland, OR, USA; 3 Providence Cancer Center, Portland, OR, USA; 4 Providence Portland Medical Centre, Portland, OR, USA

## Background

Head and neck squamous cell carcinoma (HNSCC) is the 6^th^ leading cause of cancer by incidence worldwide with approximately 600,000 new cases per year. While some patients respond to immunotherapy, the majority of patients fail to experience benefit.

## Methods

Our group formed a multidisciplinary group to study the immunobiology of HNSCC and to develop strategies to effectively treat this disease. Over the past 3 years we have collected and processed more than 192 HNSCC specimens. Starting in June of 2013 we began to set up cultures of tumor-infiltrating lymphocytes (TIL) from specimens with sufficient numbers of cells isolated by triple enzyme digestion. Between June 13, 2013 and May 14, 2015 we processed 120 specimens. Fourty-one were processed but were not set-up for TIL culture. Sixteen were determined to be grossly contaminated within 18-24 hours and excluded from further analysis. Sixty-three TIL cultures were initiated and 33 generated TIL (52%).

## Results

At this time, 22 of these have been tested for autologous tumor reactivity by IFN-γ release. Of these 22 TIL, only 2 (9%) did not exhibit autologous tumor-specific secretion of IFN-γ. An additional 3 TIL cultures exhibited low levels of IFN-γ secretion. We interpret these results to suggest that at least half of the patients in this study had mounted a T cell response against antigens expressed by their tumors. We have begun to explore the immune infiltrate and transcriptome of HNSCC and are looking for correlations between the immunoprofile, gene expression, detection of tumor-specific TIL and outcome in patients with HNSCC.

## Conclusions

We hypothesize that results of these studies could be used to stratify patients for clinical trials and ultimately tailor combination immunotherapy for patients with this disease.

